# Artificial Scaffolds in Cardiac Tissue Engineering

**DOI:** 10.3390/life12081117

**Published:** 2022-07-25

**Authors:** Jorge A. Roacho-Pérez, Elsa N. Garza-Treviño, Nidia K. Moncada-Saucedo, Pablo A. Carriquiry-Chequer, Laura E. Valencia-Gómez, Elizabeth Renee Matthews, Víctor Gómez-Flores, Mario Simental-Mendía, Paulina Delgado-Gonzalez, Juan Luis Delgado-Gallegos, Gerardo R. Padilla-Rivas, Jose Francisco Islas

**Affiliations:** 1 Departamento de Bioquímica y Medicina Molecular, Facultad de Medicina, Universidad Autónoma de Nuevo León, Monterrey 64460, Mexico; alberto.roachoprz@uanl.edu.mx (J.A.R.-P.); elsa.garzatr@uanl.edu.mx (E.N.G.-T.); pablo.carriquiryc@uanl.edu.mx (P.A.C.-C.); paulina.delgadogn@uanl.edu.mx (P.D.-G.); jdelgado.me0174@uanl.edu.mx (J.L.D.-G.); gerardo.padillarv@uanl.edu.mx (G.R.P.-R.); 2 Servicio de Hematología, University Hospital “Dr. José Eleuterio González”, Universidad Autónoma de Nuevo León, Monterrey 64460, Mexico; nidia.moncadas@uanl.edu.mx; 3 Instituto de Ingeniería y Tecnología, Universidad Autónoma de Ciudad Juárez, Ciudad Juárez 32310, Mexico; laura.valencia@uacj.mx (L.E.V.-G.); victor.gomez@uacj.mx (V.G.-F.); 4 Department of Biochemistry and Molecular Biology, University of Texas Medical Branch, 301 University Blvd, Galveston, TX 77555, USA; ermatthe@utmb.edu; 5 Orthopedic Trauma Service, University Hospital “Dr. José Eleuterio González”, Universidad Autónoma de Nuevo León, Monterrey 64460, Mexico; mario.simentalme@uanl.edu.mx

**Keywords:** biomaterials, cardiovascular diseases, cardiac tissue engineering, artificial scaffolds

## Abstract

Cardiovascular diseases are a leading cause of death worldwide. Current treatments directed at heart repair have several disadvantages, such as a lack of donors for heart transplantation or non-bioactive inert materials for replacing damaged tissue. Because of the natural lack of regeneration of cardiomyocytes, new treatment strategies involve stimulating heart tissue regeneration. The basic three elements of cardiac tissue engineering (cells, growth factors, and scaffolds) are described in this review, with a highlight on the role of artificial scaffolds. Scaffolds for cardiac tissue engineering are tridimensional porous structures that imitate the extracellular heart matrix, with the ability to promote cell adhesion, migration, differentiation, and proliferation. In the heart, there is an important requirement to provide scaffold cellular attachment, but scaffolds also need to permit mechanical contractility and electrical conductivity. For researchers working in cardiac tissue engineering, there is an important need to choose an adequate artificial scaffold biofabrication technique, as well as the ideal biocompatible biodegradable biomaterial for scaffold construction. Finally, there are many suitable options for researchers to obtain scaffolds that promote cell–electrical interactions and tissue repair, reaching the goal of cardiac tissue engineering.

## 1. Introduction

As a primary organ, the heart is fundamentally a two-sided pump responsible for maintaining blood flow throughout the body. Blood flow is kept unidirectional through a series of valves or cusps. These valves are formed from tissue folds that open and close by dilation and contraction of the heart. The smallest contractile units of the heart are cells known as cardiomyocytes (CM), primarily residing in the myocardium. CM makes up about 1/3 of the cells in the atria and about half of the cells in the ventricle [1]. Because adult cardiac cells cannot regenerate on their own, CM regeneration has been challenging for researchers to overcome [2].

### 1.1. Lack of Self-Regenerative Capabilities of Mammalian Heart Cells

Aging in the heart is accompanied by changes in vascular structure and function, especially in large arteries. While it is known that the mitotic proliferative state of human CM ceases before birth, some CM plasticity remains active over a lifetime. CM regenerative activity decreases from 1% per year (at the age of 20 years) to 0.4% (at the age of 75) [3]. This fact implies that less than 50% of CM is renewed throughout a lifetime, and about 55% of CM persists from birth [4]. The deterioration of vascular function in elderly patients is the result of phenotypic alterations of different types of cells, such as smooth muscle cells (SMC), pericytes [5], and endothelial cells (EC), that become more heterogeneous in size, shape, and axial orientation. These changes produce less laminar blood flow, increasing lipid deposition, and developing atherosclerotic plaques (cardiac ischemia) [6]. Cardiac ischemia is characterized by less oxygen flow into the heart muscle [7]. When there is ischemic injury, pathological remodeling of the heart leads to CM loss and scar formation caused by cardiac fibroblast (CF) activation. This scar formation eventually generates fibrosis and ventricle dysfunction, leading to heart failure [8]. Unlike amphibians, mammalian hearts lack regenerative capabilities to revert the fibrotic scar and replenish CM and cardiac function. The mechanisms underlying these differences are still not well understood [9]. In zebrafish, transcription factor Prrx1b promotes Neuregulin 1, which acts as a mitogen and activates CM proliferation [10]. Adult mammalian hearts exhibit minimal regenerating capacity due to a lack of a reserve population of cardiac progenitor stem cells and the almost-null proliferative ability of postnatal differentiated CM [11]. A set of chronic diseases can injure non-regenerable heart tissues and cause cardiovascular diseases.

### 1.2. Cardiovascular Diseases

Cardiovascular diseases include acute myocardial infarction, arterial hypertension, cerebrovascular disease, coronary artery disease, congenital diseases, and rheumatic heart disease [12]. Cardiovascular diseases tend to evolve gradually throughout life and are typically asymptomatic for a long time. It is estimated that by 2030, 23.6 million people per year will die of cardiovascular diseases [13]. Multiple risk factors for cardiovascular disease include genetics, smoking, physical inactivity, poor eating habits, dyslipidemia [14], type 2 diabetes, obesity, and insulin resistance. These are also associated with premature vascular and cardiac senescence [15]. It is important to mention that the most important risk factors are age and gender. Older people and women have more cardiovascular comorbidities and a higher incidence of adverse cardiovascular outcomes, including mortality after acute myocardial infarction [16].

In terms of treatment, current approaches, such as artificial vascular grafts, improve disease symptoms and slow adverse cardiac remodeling, but they fail to reverse the loss of cardiac tissue [17]. For this reason, innovative therapies, such as tissue engineering, have been proposed for their potential to stimulate the regeneration of the affected myocardium [18].

## 2. Cardiac Tissue Engineering

Delay in cardiovascular disease treatment can often create a situation in which the patient needs, for example, a valve replacement. Furthermore, heart tissue damage produced by cardiovascular diseases can lead to further heart degeneration and failure, requiring, at the extreme, full heart replacement. Sadly, donor hearts are not just in significant demand but also in very short supply. In addition, there is potential for organ rejection because of the need for tissue compatibility. Hence, the construction of specific tissue sizes and replacement heart valves would lessen the time before treatment and thus relieve the demand for full heart replacement [19,20,21].

**Figure 1:**
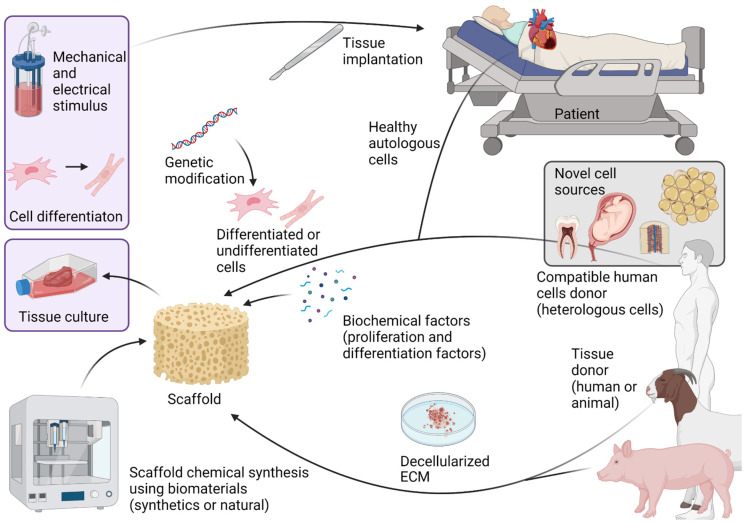
Scheme of cardiac tissue engineering development. Tissue engineering is the result of combining three basic elements: cells, growth factors, and scaffolds that imitate the extracellular matrix. Created with BioRender.com accessed on 1 June 2022.

The process of combining specific cell types, bioactive molecules (growth factors), and extracellular matrices (scaffolds) to create specific human tissues is called tissue engineering (Figure 1). The goal of tissue engineering is to replace damaged or malfunctioning tissues in human patients [1,19,22,23,24,25]. The idea of repairing an injured heart by growing cardiac cells in the lab has long been a goal of cardiac tissue engineers; however, progress to a fully functioning model has been slow. This problem is partly due to the wide variety of challenges caused by the complex structural, metabolic, and electrical demands of an organ that never stops moving. The three basic elements of tissue engineering, focused on cardiac application, are developed in this review.

## 3. Cells and Growth Factors

### 3.1. Cells

The cells that constitute the heart are CM (25–30% of heart cells); the other 70% are blood and lymphatic EC, CF, vascular smooth muscle cells (VSMC), cardiac progenitor cells (CPC), pericytes, and immune cells. CM are the contractile units responsible for pushing blood and delivering oxygen and nutrients. EC and CF are key to tissue function and homeostasis. Vascular EC are metabolically active, control vasomotor tone, and regulate angiogenesis. CFs constantly maintain the ECM through a degradation–deposition equilibrium. VSMC and pericytes regulate blood flow in the cardiac vasculature. CPC are multipotent cells with an expression of surface markers, such as c-Kit, Sca-1, MESP1+, and Isl1+. CPC comprises the stage of nascent cardiac mesoderm and can differentiate in vitro and in vivo into three major cell layers in the cardiovascular system: SMC, EC, and CM [3].

Each cell type is crucial in cardiac biology. This complexity undoubtedly raises the challenge of designing artificial heart muscle. In the last decade, great efforts have been made to obtain human cardiac lineages for various cardiac injury applications [3]. Table 1 summarizes the cell sources used in cardiac tissue engineering. Stem cells need to be stimulated to differentiate into cardiac cells for cardiac tissue regeneration.

### 3.2. Growth Factors

De novo CM in the postnatal period can be differentiated with the help of growth factors from a subpopulation of undifferentiated cardiac progenitors, or they can be transdifferentiated from CF. Both the induction of CM proliferation and CF reprogramming have granted significant benefits in preclinical experimental studies of heart regeneration. Several challenges must be overcome before this can translate into novel therapies for human heart regeneration. 

CF represents an ideal cell source for heart regeneration due to its relative abundance in this tissue. The landmark discovery that CF could be reprogrammed into a pluripotent state (iPSC) by the overexpression of different transcription factors (SOX2, KLF4, C-MYC, and OCT3/4) yielded new paradigms in cell-based therapies [37]. Ieda et al. identified a set of core cardiomyogenic transcription factors (GATA4, MEF2C, and TBX5) that induced the expression of cardiac Troponin T in mouse CF [38]. These transcription factors are part of a core set of evolutionarily conserved genes (Mesp1, Gata4, Hand2, Mef2c, Nkx2-5, and Tbx5) involved in heart development [39]. Mesp1, which is highly conserved in metazoans, is considered the cardiac master gene that drives mesendoderm differentiation via DKK1-inhibition of the Wnt/ß-catenin signaling pathway [39,40]. Downstream of the transient expression of Mesp1 comes a transcriptional network regulated by transcription factors GATA4, HAND2, MEF2C, MYOCD, NKX2-5, FOXH1, ISL1, and TBX5. This transcriptional network differentiates cell subpopulations into cardiac mesoderm lineages consisting of the first and second heart fields [39,41].

Subsequent studies using different combinations of transcription factors and gene delivery methods (for the expression of transcription factors) have successfully reprogrammed neonatal and adult fibroblasts into cardiomyocyte-like cells in experiments both in vitro and in vivo [8,42]. GATA, MESP1, and TBX5 (GMT) overexpression has been shown to induce the expression of sarcomere structures in fibroblasts; moreover, the addition of HAND2 markedly improves reprogramming efficiency to CPC and enhances the function of injured hearts following myocardial infarction [42]. CPC maintenance and differentiation are regulated via NOTCH and WNT signaling [39]. NOTCH inhibition substantially increases MEF2C binding to its target genes, producing cardiac differentiation (in cell clusters) marked by a rise in calcium flux and beating colonies [43]. BMP and WNT signaling inhibition differentiate CPC into a myocardial lineage marked by the expression of cardiac isoform troponin T and the expression of the transcription factor NKX2.5 [40,44]. The combination of the histone deacetylase inhibitor sodium butyrate, the WNT inhibitor ICG-001, and the cardiac growth regulator retinoic acid has been shown to enhance cardiomyocyte-like cell generation in rat CF [45]. Other strategies for cell differentiation implicate cell mRNA degradation using miRNAs. The combination of miR-1, miR-133, miR-208, and miR-499 induces the expression of the αMHC-CFP reporter in 1.5 to 7.7% of neonatal CF. In vitro and in vivo administration into ischemic mice myocardium induces conversion from CF to CM [46]. Delivery of miR-106b~25 into mouse hearts produces CM proliferation by targeting a network of negative cell cycle regulators, including E2f5, Cdkn1c, Ccne1, and Wee1. Proliferation produces almost complete regeneration of the adult myocardium after ischemic injury [47]. Three different miRNAs (miR-548c-3p, miR-509-3p, and miR-23b-3p) were identified as inhibiting the posttranscriptional activity of the antimitotic gene Meis1, inducing CM proliferation [48].

Another novel strategy that has shown good results in the use of factors for the regeneration of cardiac tissue is the use of a cell secretome. Stem cells secrete a combination of different molecules such as cytokines, growth factors, enzymes, microvesicles/exosomes and genetic material. It has been demonstrated that this cell secretome helps in cardiac tissue regeneration, inducing CM survival, proliferation, differentiation, neovascularization, and limiting inflammatory and profibrotic processes [49].

## 4. Scaffolds

**Figure 2:**
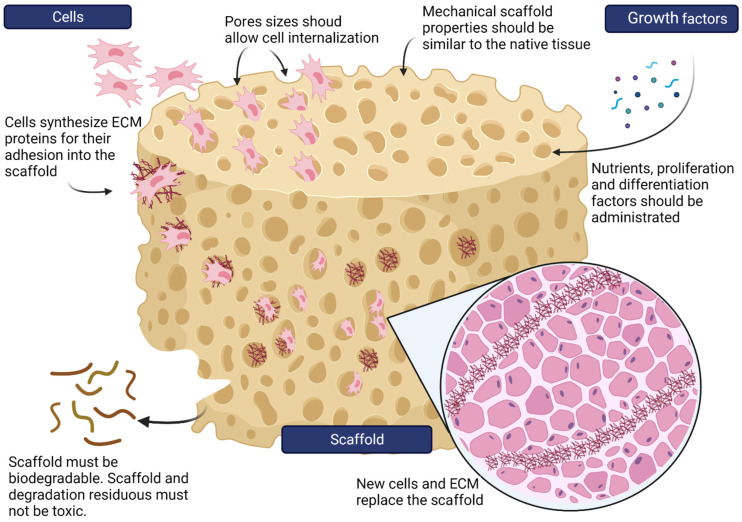
Scheme of the principal properties of scaffolds. Created with BioRender.com accessed on 1 June 2022.

Scaffolds are tridimensional porous structures prepared with biocompatible and bioactive materials, with the ability to promote cell adhesion, migration, differentiation, and proliferation in vitro and in vivo (Figure 2) [1,50].

All these scaffold properties are possible because scaffolds are designed for extracellular matrix (ECM) imitation. The ECM is essential for cells to connect and communicate with each other, providing tissue structure organization and intercellular signaling [51]. In brief, all scaffolds used in tissue engineering must have the following essential properties:

### 4.1. Porosity

The porosity is related to the amount of pore space in the scaffolds. Some physical properties, such as material density, can be used to calculate scaffold porosity [52]. Scaffolds need to have appropriate porosity through pore interconnection, appropriate pore size, and pore size distribution [49]. Pores allow cell migration and facilitate nutrient supplementation [1,49]. Scaffolds should have 50–90% permeability to promote the diffusion of nutrients, oxygen, and other fluids [53].

### 4.2. Topography

Scaffold topography is defined as the surface spatial features of a 3D scaffold. This property is important because different cellular responses to different topographical cues have been reported in in vitro and in vivo studies. The detailed mechanisms of these interactions have yet to be elucidated. The cellular responses to scaffold different topographies include cell adhesion, cell migration, and changes in cell morphology. These responses affect the growth, differentiation, and proliferation of cells [54].

### 4.3. Surface Functionalization

To improve cell adhesion to the scaffold surface, it is necessary to perform changes in the surface chemistry and physical topography of the scaffolds. This change (functionalization) can be performed by adding atoms or molecules to the surface of the scaffold. This functionalization can be achieved through physical or chemical methods. A highly used strategy for scaffold functionalization is to coat the scaffold with bioactive molecules all over the scaffold surface. Physical functionalization consists of a weak interaction between the scaffold ligand and the bioactive molecule through weak electrostatic interactions, hydrogen bonding and/or hydrophobic interactions. A disadvantage of physical functionalization is the lack of control over the orientation of functionalized bioactive molecules. In the other hand, chemical functionalization involves covalent bond formation, which means a stronger oriented interaction [55].

### 4.4. Mechanical Properties

Scaffolds mimic ECM properties, giving cells mechanical support for proliferation and tissue formation. The mechanical and structural properties of scaffolds need to be similar to the mechanical properties of the tissue that will be repaired [1,50]. Fiber length is an important parameter for mechanical improvement. When fibers are incorporated, the strength and stiffness of the scaffold can be improved. The effects of 2 mm fibers were up to three times greater than those of 10 mm fibers at identical mass ratios [56,57,58].

### 4.5. Electrical Properties

Electrical behavior is an important scaffold property in cardiac application. The cardiac muscle is an electroactive tissue that can transfer electrical signals across the heart. Cardiac tissue engineering requires materials that can provide a similar bioelectronic interface. Conductive materials for preparing scaffolds are preferred in cardiac regeneration. Nonconductive materials can improve their electrical potential by functionalizing with conductive materials, such as carbon nanotubes, carbon nanofibers [59], or graphene oxide [60].

### 4.6. Biodegradation

Scaffolds must be biodegradable to gradually disappear while cells generate their own ECM and replace the scaffold. Residual molecules generated from biodegradation should be eliminated easily from the body, or they can be integrated into different celllular metabolic routes [1,50].

### 4.7. Bioactivity

Scaffold surfaces have the property of bioactivity, which means that the scaffold surface interacts with the biological media, resulting in the formation of a bond between the cells and the scaffold. In addition, scaffolds can interact with tissues, including cells and extracellular matrices [50,61].

### 4.8. Non-Toxic

Materials chosen for scaffold construction should be non-toxic or immunogenic. This property also applies to the residual molecules generated after degradation [1].

## 5. Decellularized Extracellular Matrices

As an alternative to artificial scaffolds, decellularized extracellular matrices are a natural source for scaffolds. Decellularization is the total elimination of cells from tissue by physical or chemical methods. After cell removal, the obtained scaffold consists of natural basic structural ECM proteins and glycosaminoglycans [62]. The lack of cellular components eliminates the risk of adverse side effects, such as an inflammatory response or immunological rejection. If the obtained 3D structure is not warranted, matrices might pass through drying and pulverization for further reconstitution into molds or shapes of interest [63,64,65].

Data from preclinical and clinical evaluation of acellular biologic ECM scaffolds in cardiac pump dysfunction and ischemic heart failure showed reduced fibrotic tissue, improved perfusion of infarcted myocardium, and reverse structural remodeling [66]. A decellularized pericardial matrix colonized with human viable Wharton’s jelly-derived mesenchymal stromal cells was implanted in patients with non-revascularizable myocardial scars and has shown a reduction in scar mass after three months [67]. A previous study using human decellularized pulmonary heart valves engineered with autologous EPC in pediatric patients with pulmonary valve pathology reported that these valves have the potential to remodel and grow along with the somatic growth of the patient [68].

A potential disadvantage of decellularized extracellular matrices is the existence of remaining cells from the native tissue in the scaffold. These remaining cells can be dangerous if they trigger a patient’s immune response [62]. In addition, an alternative to decellularized extracellular matrices is the use of artificial scaffolds.

## 6. Biofabrication Approaches Used to Develop Artificial Scaffolds

**Figure 3:**
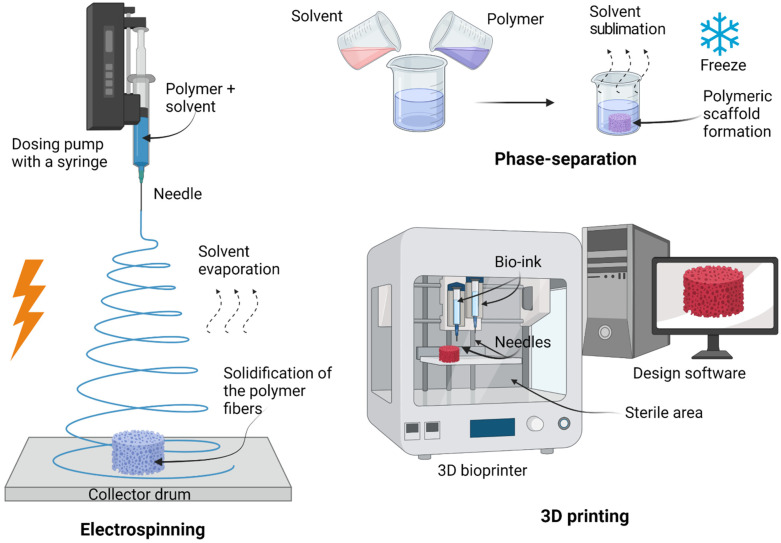
Scheme of principal preparation techniques for scaffolds. Electrospinning consists of the formation of fibers using a voltage source. This involves the generation of a charged polymer jet that is ejected through a high-voltage. 3D printing generates a scaffold using computer design software, printing the scaffold layer by layer. Created with BioRender.com accessed on 1 June 2022.

Different techniques are used to create artificial scaffolds to improve their physical and biological properties [69]. Artificial biodegradable scaffolds hold the inherent advantage of being easily crafted into any shape and engineered for desired mechanical properties [65]. Various techniques have been developed to fabricate artificial scaffolds that mimic the ECM-native myocardium. These include, but are not limited to, electrospinning, phase separation, and 3D printing (Figure 3) [69]. Understandably, each technique has its advantages and disadvantages, which we will discuss in general from the most commonly used artificial scaffold biofabrication methods, in this section.

### 6.1. Electrospinning

Electrospinning is a technique used for micro- and nanoscale fiber production from an electrically forced solution/melt of polymeric biomaterials [70]. The four basic elements required to implement electrospinning are a dosing pump with a syringe (containing the polymer solution), a needle (Taylor cone), a collector drum (can be a plate, metal screen, or rotating mandrel), and a high-voltage power supply (up to 30 kV). Briefly, electrospinning involves the generation of a charged polymeric biomaterial jet that is ejected through a high-voltage electric field; randomly rotating polymeric fibers rest on a grounded complex to create a scaffold. The solvent evaporates, and the polymer fibers solidify [71,72]. It is important to consider every single parameter, polymer molecular weight, voltage, the distance between the capillary and collector, polymer concentration, solution conductivity, and solvent volatility, all of which impact the characteristics and properties of the fibers [73]. An important parameter in cardiac tissue engineering is the diameter of the fiber. The diameter of the fiber in electrospinning plays an important role in the conductivity and properties that are needed in cardiac regeneration. According to Abedi et al., the reduction of average fiber diameter from 225 to 110 nm in a scaffold based on chitosan and PVA with multi-wall carbon nanotubes increased electrical conductivity from 8 × 10^−5^ S/m to 9 × 10^−3^ S/m [74].

- Advantages: simple methodology, relatively low preparation cost, sample uniformity, generation of aligned fibers smaller than a micrometer, scaffold incrementation of surface-to-volume ratio (it improves cell attachment, proliferation, and differentiation), tunable porosity interconnectivity over 80%, unique pore shapes, and superior mechanical properties [70,71,75,76];
- Disadvantages: the need for high-voltage appliances and the use of toxic solvents [76].

### 6.2. Phase-Separation

Phase separation, also called thermally induced phase separation, is a simple method that comprises solution preparation with polymeric biomaterials and solvents, followed by freezing. Once the mix is prepared, the solvent is removed without degrading the polymer by sublimation via freeze-drying to obtain scaffolds with high porosity and interconnectivity [77]. Freezing temperature, concentration, and the nature of solvent and solute can be tunable to modify some pore characteristics [78].

- Advantages: simplicity of the technique, and preservation of the scaffold structure because the process does not involve high temperatures [76];
- Disadvantages: lengthy process, poor architecture, limited control of size range, irregular porosity, unsuitable mechanical properties for replacing human tissue, and remaining solvents are potentially toxic [76].

### 6.3. 3D Printing

Another novel method for fabricating artificial scaffolds is 3D printing. Its use involves bio-printers, needles, bio-inks, and design software [79]. A 3D model was generated using computer-aided design (CAD) software for conversion to STL format. This 3D format of the scaffold is sliced into 2D layers to be successively printed and bound layer by layer. Hence, the resolution level depends on the diameter of the needles used [80,81]. Some current bio-printer models mimic a sterile environment, such as a biosafety cabinet, using HEPA filters and UV lamps for sterilization. This process can be observed because of their clear windows [72].

- Advantages: probably the most attractive technique in terms of micro-architecture, allows the use of a wide range of biomaterials, and excellent controllability over structural properties such as porosity, pore size, and pore interconnectivity [79];
- Disadvantages: initial investment in the instrument, the use of toxic solvents, and mechanical instability [72].

### 6.4. Solvent Casting and Solvent Casting/Particulate Leaching

The solvent casting method is based on polymer and organic solvent mixing, casting into a 3D mold, or immersing the mold in a polymeric solution to get a scaffold once the solvent has been removed by simple evaporation, vacuum drying, or lyophilization. After solvent removal, the scaffold is washed with water, leaving the porous structure. The internal size of pores is related to and controlled by salt granules [76,78,82].

- Advantages: simple methodology, mechanically stable, and does not need sophisticated instruments [76,78,82];
- Disadvantages: maintaining desired porosity and uniform salt dispersion is a challenge, a long time for solvent evaporation, inefficient complete salt leach out, and inefficient complete removal of the solvent [76,78,82].

### 6.5. Emulsion Templating

- Emulsion templating is a promising biofabrication approach for scaffolds with up to 99% porosity and high interconnectivity. The technique is based on two steps. The first step is the preparation of an emulsion of two immiscible liquids; one phase is the internal or dispersed phase, and the other one is the external or continuous phase. The second step is the solidification of the continuous phase of the emulsion, while the dispersed phase (which works as a template by its addition drop-by-drop) is removed to obtain a porous scaffold. Emulsion can be given by water-in-oil (*w*/*o*) or oil-in-water (*o*/*w*) [83].

## 7. Materials Used for Artificial Scaffold Construction

Biomaterials can be defined as any material or combination of materials that can be used for any period for the total or partial replacement of any tissue. Biomaterial’s goal is to maintain or improve the quality of a patient’s life. Polymeric biomaterials can be classified by their synthetic or natural origin [84]. Polymeric biomaterials are an attractive option for tissue engineering since they can monitor their development, thus controlling topological, mechanical, and structural properties with the benefit of functionalizing or adding other materials (composites) to enhance attachment [85,86]. Over time, many scaffold formulations have been developed by mixing two or more different biomaterials to mimic the ECM native myocardium and its mechanical and biological properties [87].

### 7.1. Synthetic Materials for Artificial Scaffolds

Although not all synthetic materials are directly biodegradable, non-biodegradable biomaterials can be engineered to biodegrade. Biodegradable materials have a great advantage. They have the capability to be replaced when mature cells produce their own ECM. Using synthetic materials in cardiac tissue engineering has grown in recent years, with emphasis on developing patches for cardiac repair with the use of biodegradable polymers, such as poly(ε-caprolactone) (PCL), poly(glycerol sebacate) (PGS), polyethylene glycol (PEG), poly(l-lactide acid) (PLLA), poly l-lactic-co-ε-caprolactone (PLCL), and poly(lactic-co-glycolic acid) (PLGA) [53]. In the early part of the 21st century, Matsubayashi et al. developed a porous PCL cardiac patch seeded with VSMC with the intent of repairing an aneurysm and designed to prevent ventricular dilation post-myocardial infarction [88]. Moreover, Morgan et al. developed other porous structures using PGS to control the orientation of cardiac cells [89]. Improving cardiac orientation, Hu et al. developed patches using PGS copolymerized with an aniline trimer to obtain electroactive material, significantly enhancing cell interactions [90]. An interesting part of heart mending is valve repair, wherein materials such as PEG-based hydrogels are used as scaffolds. By cross-linking PEG with peptides, biomimetic properties were obtained; the resulting hydrogels influenced elongation. The de novo ECM deposition and hydrogel degradation behavior of encapsulated valvular cells potentiated the use of these materials for developing future heart valves [91]. In another interesting approach, the implantation of tissue-engineered vascular grafts (PLLA and PLCL) seeded with autologous bone marrow mononuclear cells was used as an extracardiac total cavopulmonary conduit in pediatric univentricular physiology. No evidence of aneurysmal formation, graft rupture, graft infection, or calcification was reported, and seven (28%) patients had asymptomatic graft stenosis [92].

### 7.2. Natural Biomaterials for Artificial Scaffolds

In the search for natural biomaterials, researchers have investigated the individual components of the ECM as platforms. Many biomaterials for scaffold construction, such as collagen, fibrinogen, silk, alginate, and chitosan, are under investigation [53,93,94,95,96].

#### 7.2.1. Collagen

Collagen is commonly used in myocardial tissue engineering, given that it is a major component of the myocardium ECM [97] (Table 2). In the heart, collagen type I is the major constituent of the ECM, representing 75–85% [98], with the added advantage of low immunogenicity. Collagen type I comprises two alpha-1 chains and one alpha-2 chain, creating long fibers whose properties depend on density and spatial alignment [85], albeit collagens can divide into fibrillar and non-fibrillar components. These non-fibrillar components can form networks or associate with fibrillar collagens or membranes [99,100]. Recent research has focused on utilizing collagen-based biomaterials for pathologies, such as myocardial infarction. These materials can deliver particulates, such as growth factors or peptides, to induce differentiation and patterning [101]. Researchers have based initial approaches to delivering these materials on intramyocardial injection, as this permits direct and precise delivery to the affected area. However, with this approach, surgery becomes a must, and there is a second issue of potential leaking of the material to other areas [102]. An alternative to this delivery system is the formation of so-called “cardiac patches”. Even though they are not exclusive to collagen, cardiac patches have unique properties. One such feature is their potential to cultivate cells ex vivo to promote proper invasion of the patch; further, these patches can go into models and have high engraftment levels [93]. Lastly, a 3D collagen type I matrix seeded with autologous bone marrow mononuclear cells (for the regeneration of ischemic myocardium) showed increased thickness of the infarct scar with viable tissue and helped normalize cardiac wall stress in injured regions, limiting ventricular remodeling and improving diastolic function [103].

#### 7.2.2. Fibrinogen

Previous work has used collagen and fibrin/fibrinogen patches to enhance cardiac cell maturation by simulating cardiac muscle development [93,94]. One clear advantage of these materials is their help in promoting electrical conductivity, particularly in differentiating cells and other stimulants. Certain maturation properties are achieved, such as the induction of Purkinje-like cells [93,108,109]. Fibrin is a naturally occurring biomaterial, a biopolymer formed during coagulation [98,110]. It has interested many researchers, either alone or in combination. Fibrin/fibrinogen helps with certain properties, including biocompatibility and biodegradability, when used as a scaffold. Fibrin comprises a randomly organized 3D structure with high interconnectivity, yet the threads forming the network are soft, allowing deformation without a break. Fibrinogen decomposition (fibrinopeptides), which depends on the amount of thrombin used and re-polymerization, regulates the mechanical properties of fibrin gels [110]. In clinical applications, fibrin is obtained from plasma for autologous applications, such as treating osteoarthritis [96]. It is important to mention that fibrin glue is another important application typically used in surgical procedures to replace sutures [111]. Other useful applications include repairing the urinary tract, eye, liver, lung, spleen, heart valves, and filling bone cavities [98,111,112,113].

#### 7.2.3. Silk

The use of silk as a novel biomaterial for tissue engineering has been studied in recent years (Table 3). Silk has been studied not only because it has several similarities to other materials, such as fibrin/fibronectin (architecture, mechanical properties, and degradation rates) but also because silk, in comparison with fibronectin, does not contribute to pathological hypertrophy [114]. In animal models, silk-based scaffolds have shown therapeutic effects and the capacity to maintain the differentiation of cells into cardiac lineages [115]. It is important to note that orientation is key, as defined in cardiac tissue studies, to demonstrating the maintenance and development of sarcomeres, particularly an upregulation in the synthesis of titin protein [116].

#### 7.2.4. Alginate

Alginate is a natural derivative of the cell walls of brown marine algae or bacteria. It is a polysaccharide with properties of biocompatibility, solubility in postmodified salts or esters, porosity, biodegradability, and viscosity tunability. Besides their use in tissue engineering, alginate has been used in medical applications, such as drug and protein delivery, wound healing, and implants. Disadvantages in the medical use of alginate in cardiac tissue engineering include inadequate mechanical stability and poor biological stability (inhibition of proliferation and instable biodegradability). As an option for solving these disadvantages, composite scaffolds are preferred by the combination of alginate with other polymers (Table 4) [118].

#### 7.2.5. Chitosan

Chitosan (CS) is a polysaccharide obtained by the deacetylation of chitin. Normally found in insects and crustaceans, such as shrimp, crabs, and lobsters. It is a natural polymer with a linear structure consisting of β (1–4) glycosidic bonds linked to d-glucosamine residues with a variable number of randomly located N-acetyl-d-glucosamine (NAG) groups. It is soluble in dilute or weak acids but insoluble in aqueous solutions above pH 6.5 [99]. Recently, chitosan has been widely used in tissue engineering because of its capacity to protonate amino groups in an acidic medium, providing high biocompatibility, non-toxicity, anti-thrombogenic, biodegradability properties, and possessing a hydrophilic surface [120]. The combination of chitosan scaffolds with stem cells has shown positive results in delivering stem cells to infarcted myocardium and increasing cell retention while preserving cardiac function [121]. These properties make chitosan scaffolds suitable for cell attachment and proliferation (Table 5). However, chitosan scaffolds alone have limitations because they have weak mechanical properties and a high degradation rate [50].

There is no evidence of the clinical use of chitosan-based tissue engineering therapies. Nevertheless, recent research has been devoted to studying the potential use of chitosan-based biomaterials as an injectable therapy delivering progenitor cells [124]. Because of its hemostatic properties, chitosan has been used in chitosan-based pads to improve hemostasis following transradial arterial access in a couple of clinical studies [125,126] and a few more ongoing clinical trials (NCT04380883, NCT04857385, NCT03522077, NCT02837744).

## 8. Composite Artificial Scaffolds

As shown in Table 2, Table 3, Table 4 and Table 5, actual investigations focus on generating scaffolds not only using one biomaterial, but also in the use of composites, generating new biomaterials with the benefits of both materials chosen for the composite fabrication. A notable example is the use of chitosan. To improve the properties of chitosan scaffolds, researchers have carried out many studies to create chitosan composite scaffolds, which means chitosan combined with other polymers, including collagen [99], PLGA [127], polyurethane [128,129], PLLA [130], PLA [131], PEG [132], or PCL [133]. The composite’s goal is to improve the mechanical, electrical, and biological properties of the scaffold. Even non-polymeric aggregates have been effective for enhancing chitosan scaffolds, such as carbon nanotubes [134], or graphene [135,136]. Carbon nanostructures are commonly used to improve electrical conductivity. They facilitate the electron transfer of electroactive analytes. In addition, carbon nanofibers can further reinforce polymer scaffolds and produce excellent mechanical properties [59,136].

Collagen/chitosan composites are known for their physical characteristics, biocompatibility, and contractile performance [99]. Another particularity of chitosan scaffold improvement is the addition of PLA by conventional electrospinning. Adding PLA fibers to chitosan significantly improved both elongation and tensile strength, increasing Young’s modulus from 57.38 ± 13.21 MPa to 78.67 ± 14.15 MPa. This large increase is attributable to the thermodynamic immiscibility and inherent incompatibility between thermoplastic polymers and chitosan; thus, composite scaffolds consisting of PLA have great potential for cardiac tissue enhancement and regeneration [130]. Polyurethane/chitosan/CNT nanofibrous scaffolds have shown appropriate biocompatibility to support cell attachment and proliferation, demonstrating the potential of these scaffolds in cardiac patch applications [129]. Polycaprolactone/gelatin/chitosan hydrogel scaffolds with excellent biodegradable and tensile strength properties can be used to develop patches with the potential for surgical reconstruction of congenital heart defects [124]. Chitosan/ polyvinyl alcohol/ multiwalled carbon nanotubes scaffolds have also shown positive results with similar properties in the cardiac tissue differentiation process [74]. According to Liu et al., a cardiac patch loaded with TiO_2_ nanoparticles into a PEGylated chitosan hydrogel scaffold enhances physicochemical, mechanical, and biological properties [132]. Biodegradable chitosan-based patches have also been developed with the potential for the surgical reconstruction of heart defects. One such example can be seen in the work by Pok et al., wherein they developed a hydrogel based on a PCL self-assembled sandwiched in gelatin–chitosan. Their biomaterial mixing procedure allowed optimal suturing properties due to PCL tensile strength; on the other hand, the gelatin–chitosan scaffold allows cell attachment and mechanical properties that promote CM migration and function [133].

## 9. Conclusions

Undoubtedly, there are multiple models and proposals for artificial scaffolds, in combination with cells and growth factors, to provide an adequate microenvironment to mimic the characteristics necessary for the maintenance of the cardiac phenotype: the structural, mechanical, and conductivity properties of the native tissue. Mimic of native tissue properties, such as electrical conductivity and mechanical contraction properties, has been possible with the correct choice of the used material and the biofabriocation approach. Another main point in the development of artificial scaffolds for cardiac tissue engineering is the elaboration of easy-construction scaffolds. Scaffolds should be developed with low-cost materials. They should be easily sterilized and chemically stable, with a long storage life, and their mass production should be scalable from the laboratory.

We have a long road ahead to develop the perfect scaffold, expand the knowledge on cell–electrical interactions, promote electroactive tissue repair, and finally generate artificial hearts. Nevertheless, studies published in the last few years provide us information about the novel properties and techniques that stem cells, growth factors, and scaffolds should have, providing positive insights into the potential of cardiac tissue engineering, and reaching the goal of medicine and tissue engineering.

## Figures and Tables

**Table 1:** Cell sources in cardiac tissue engineering.

Source	Cells	Definition	Advantages	Disadvantages	Reference
Embryonic	Fetal CM	Fetal heart.	Potential for cardiac integration and regeneration.	Immunogenicity. Malignant potential. Ethical questions. Limited availability.	[26]
	Human umbilical cord blood-derived cells	Pluripotent stem cells, mesenchymal stem cells (MSC), hematopoietic stem cells (HSC), and non-hematopoietic stem cells (NHSC). Differentiate into different types.	Reduction of infarct after intramyocardial injection.	Immunogenicity. Need to standardize isolation and culture procedures. Senescence and mutational acquisition during in vitro expansion.	[27,28]
	Embryonic MSC	Pluripotent stem cell derived from inner cell mass of blastocyst in embryo.	Potential to differentiate into cells from all three embryonic germ layers.	Associated with malignant transformation. Legal issues.	[29]
Adult stem cells	Adipose stem cells	Derived from adipose tissue: heterogeneous mixture of MSC, HSC, and endothelial progenitor cells (EPC).	Multipotent potential. Easily source with minimal effort. Easy harvesting. Low cost. No ethical issues.	Potentially tumorigenic. Limited understanding of the mechanism associated with cardiac repair.	[30]
	Bone marrow stem cells	Stem cells derived from bone marrow.	Well-known cell precursors. Easy collection.	Extracted in low numbers. Potential contamination during in vitro expansion.	[30,31]
	EPC	Originated from different tissues. Have been classified into hematopoietic and nonhematopoietic progenitor cells.	Increase its numbers in response to ischemia/cytokine stimuli. Migrate to injury site and differentiate into myocytes. Participate in repair and maintenance of vascular homeostasis.	Low numbers in peripheral blood and bone marrow makes ex vivo expansion difficult.	[32]
	Resident cardiac stem cells (RCSC)	Represent a responsive stem cell reservoir within the adult associated myocardial homeostasis.	Capable of differentiating into multiple cell types such as CM or VSMC.	Limited repair.	[33]
Ault somatic cells	Human-induced pluripotent stem cells (iPSC)	Autologous somatic cells that can be converted into pluripotent cells.	Possibilities of large-scale production. Ability to differentiate into CM, SMC, and vascular EC.	Poor purity. Heterogeneity. Laborious/inefficient techniques of isolation. Can generate teratomas.	[30,34]
	CF	Source of induced pluripotent cells. Can be reprogrammed directly into CM, EC, and SMC.	Available in large numbers. Phenotypically plastic. Promote the proliferation of endogenous CM by activation of the CM cell cycle.	Primary drivers of fibrosis. Unclear how an in vivo environment with changed ECM compositions influences CF plasticity and integration.	[35]
	Skeletal myoblasts	Derived from muscle biopsy.	Ability to engraft, create myotubules, and improve cardiac function after transfer into infarcted myocardium.	Heterogeneous. Associated with arrhythmias, interfering with the propagation of electrical potentials.	[36]

**Table 2:** The use of collagen for scaffold fabrication in cardiac tissue engineering.

Scaffold	Biofabrication Technique	Results	Year	Reference
Conductive nanofiber scaffold (polypyrrole hidrogel/chitosan/polyethylene oxide)	Electrospinning	Cell adhesion, growth and proliferation, conductive nanofiber scaffolds appropriate for employing in body parts with electrical signals such as cardiac tissue engineering.	2021	[104]
Collagen/chitosan composite scaffold	Freezing and lyophilization	High porosity (>65%), excellent mechanical properties, in the physiological range of native myocardium, biocompatibility, CM high expression of cardiac-specific marker protein, and contractile performance.	2020	[105]
Injectable hidrogel (Collagen/carbon nano tubes/chitosan/gold nanoparticles)	Chemically cross-linking	Non-toxicity, great potential as a new biomaterial for cardiac tissue engineering applications.	2020	[106]
Collagen/graphene oxide cardiac patch	Freeze-drying method	Interconnected pores with appropriate pore sizes, electrical conductivity suitable for cardiac tissue engineering, no toxic effects on human cells, neonatal cardiomyocyte adhesion, and upregulation of cardiac genes expression.	2019	[107]

**Table 3:** The use of silk for scaffold fabrication in cardiac tissue engineering.

Scaffold	Biofabrication Technique	Results	Year	Reference
Polypyrrole scaffold coated with silk fibroin	Electrospinning	Mimic of myocardium fibrils, similar mechanical properties to the native myocardium, sufficient electrical conductivity for cardiomyocytes, support CM contraction.	2021	[117]

**Table 4:** The use of alginate for scaffold fabrication in cardiac tissue engineering.

Scaffold	Biofabrication Technique	Results	Year	Reference
Alginate scaffolds functionalized with magnetite nanoparticles	Freeze-dry technique	Magnetic alginate scaffolds exposed to an alternating magnetic field create stimulating microenvironments for engineering functional tissues	2021	[119]
Composite of cardiac ECM with alginate and chitosan	Freezing and lyophilization	Porosity of more than 96%, very high swelling rate, stability in PBS solution, improving of the tensile strength, proliferation of human MSC inside the pores, higher expression of cardiac marker cTnT.	2020	[120]

**Table 5:** The use of chitosan for scaffold fabrication in cardiac tissue engineering.

Scaffold	Biofabrication Technique	Results	Year	Reference
Polyurethane /CS/carbon nanotubes composite	Electrospinning	Biocompatibility, electro conduction, and aligned nanofibers.	2021	[122]
CS scaffold blending with graphene oxide (GO)	Freezing and lyophilization	Swelling, porosity, and conductive properties. Good cell viability, promotion of cell attachment, intercellular network formation, upregulation of cardiac-specific genes, and protein expression involved in muscle conduction of electrical signals.	2019	[62]
Cardiac ECM-chitosan-gelatin composite	Freezing and lyophilization	High porosity, biodegradable and biocompatible scaffold, increased cell survival and proliferation, promotion of differentiation process.	2019	[123]

## Data Availability

Not applicable.

## Abbreviations

CF	Cardiac fibroblast
CM	Cardiomyocytes
CS	Chitosan
CPC	Cardiac progenitor cells
EC	Endothelial cells
EPC	Endothelial progenitor cells
ECM	Extracellular matrix
GO	Graphene oxide
HSC	Hematopoietic stem cells
iPSC	Induced pluripotent stem cells
MSC	Mesenchymal stem cells
NHSC	Non-hematopoietic stem cells
PCL	Poly(ε-caprolactone)
PDMS	Polydimethylsiloxane
PEG	Polyethylene glycol
PGS	Poly(glycerol sebacate)
PLA	Polylactic acid
PLCL	Poly-l-lactic-co-ε-caprolactone
PLGA	Poly(lactic-co-glycolic acid)
PLLA	Poly(l-lactide acid)
PVA	Polyvinyl alcohol
RCSC	Resident cardiac stem cells
SMC	Smooth muscle cells
VSMC	Vascular smooth muscle cells
